# Avatars using computer/smartphone mediated communication and social networking in prevention of sexually transmitted diseases among North-Norwegian youngsters

**DOI:** 10.1186/1472-6947-12-120

**Published:** 2012-10-30

**Authors:** Elia Gabarron, J Artur Serrano, Rolf Wynn, Manuel Armayones

**Affiliations:** 1 NST-Norwegian Centre for Integrated Care and Telemedicine, University Hospital of North Norway, P.O. Box 35, N-9038, Tromsø, Norway; 2 Department of Clinical Medicine, Faculty of Health Sciences, University of Tromsø, Tromsø, Norway; 3 Division of Addictions and Specialized Psychiatry, University Hospital of North Norway, Tromsø, Norway; 4 PSiNET Research Group, Internet Interdisciplinary Institute (IN3), Open University of Catalonia, Barcelona, Spain

**Keywords:** Sexually transmitted diseases, Chlamydia, Health information technologies, Internet, Health education, Social network, Social media, Young adult, Adolescent

## Abstract

**Background:**

Sexually transmitted diseases (STDs), especially the *Chlamydia trachomatis* bacterial infection, a common cause of infertility, are highly prevalent in developed countries, and a worrying problem in North Norway, where the incidence of chlamydia twice the Norwegian average. Seventy percent of reported chlamydia cases are found in people below 25 years of age, and although its spread could be controlled with proper prevention, young people are more aware of the risks of unwanted pregnancy than their risk of acquiring a STD. Information and Communication Technologies, including, the Internet, social media and/or smartphones, should be valued for sexual health promotion for their potential to engage young audiences. And in these media, avatars guarantee anonymity to users when handling sensitive information. The main objective of this project is to achieve that North Norwegian youngsters become more aware of STDs through the use of popular technologies among young people.

**Methods:**

A Virtual Clinic for Sexually Transmitted Diseases (VCSTD) will be developed. The VCSTD will provide early guidance and reliable information sources concerning reproductive health, delivered in a novel and innovative way to the younger population. The VCSTD consists of an “avatar” supported intervention in a serious gaming and e-learning environment, which will bypass direct physical access (in person) to reliable medical information, as well as allowing the youngsters to share that information in social media, and thus helping the VCSTD to be disseminated to more people.

Data analyses will be conducted on publically available health data relevant to STDs in Troms and Finnmark, like the absolute number of chlamydia tests, the amount of emergency contraception medication sold, and the number of abortions. Also, usage data of the system and experiences of usefulness will be explored through participants’ voluntary responses to a feedback form available in the VCSTD.

**Discussion:**

This study will examine the usefulness of an online public health intervention that aims to promote healthy sexual practices among North-Norwegian youngsters. If shown to be effective, the intervention could prove to be an affordable and widely accessible intervention to decrease risky sexual practices in younger population.

## Background

It is well known that a young age at the first experience of intercourse, a poor condom use and an increase in the number of lifetime sexual partners favour the transmission of sexually transmitted diseases (STDs) [1], with possible serious medical an social side effects with considerable consequences to the lives of the affected (i.e., unwanted pregnancies, infertility, AIDS…).

Every year, approximately 90 million new cases of genital *Chlamydia trachomatis* infection (hereafter referred to as "chlamydia") are diagnosed worldwide [2]; and this infecting pathogen is the most commonly responsible for STD according to the World Health Organization [3]. The most severe consequences of chlamydia affect women, and although many of these infections remains asymptomatic [1], pelvic inflammatory disease occurs in 30% of the untreated women and approximately one third of these women will become infertile, have ectopic pregnancies or develop chronic pelvic pain, leading to an increased risk of cervical cancer and pneumonia of the newborn [2].

Chlamydia, with a mean incidence rate of 467 cases per 100.000 inhabitants in 2009 [4] is the most common reportable sexually transmitted infection in Norway. And the two northernmost counties, Troms and Finnmark, with a population of 155.061 and 73.787 [5] have had the highest chlamydia incidence rates in Norway, with 684 and 898 respectively [4]. Not enough, 70% of reported chlamydia cases are found in people below 25 years of age [6]. Analyses of chlamydia test numbers in Norway have also shown that the frequency of testing is low in the youngest age group, which is at high risk of infection [7].

### Promotion of safer sex practices through ICT

Young people generally lack knowledge about sexually transmitted diseases and are more aware of the risks of unwanted pregnancy than their risk of acquiring a STD [1]. In view of the situation, the Information and Communication Technology (ICT) could be a good way to achieve that youth become more aware of STDs, because Norway has one of the highest Internet penetration rates in the world (97.2%) [8] and these media are among the most powerful influences in the lives of young people [9]. A Norwegian survey performed in 2^nd^ quarter 2011 found that 100% of youth from 16 to 24 years old had been using Internet in last 3 months, 93% of them use Internet every day or almost every day; and 96% of activities on Internet were related with social networks, as Facebook, Twitter, etc [10].

The use of ICT related with sexually transmitted diseases has been studied during last recent years [11,12]. Now we have a greater knowledge about the important role of Internet on STD knowledge and sex education for young people [1] and we can say that although the broad dissemination of personal computers, smartphones, Internet access, and social networking sites, particularly in developed countries, has created a new ‘risk environment’ in which potentially STD-infected sex partners meet, but also an intervention can occur for conducting promotion of safer sex [11,13,14]. A bibliographic review performed by Swendeman and Rotherman-Borus shows the efficacy of computer-based, Internet-based and mobile phone behavioural for STD prevention and treatment support interventions [14]. The authors found that ICT can transform targeted, routine, and consumer-controlled sexually transmitted disease testing as well as the partner intervention [14].

A more recent study with young people aged 15 to 24 from Vancouver found that, in general, youth (age<25) are particularly receptive to internet-based services [15]. Youth in this study suggested that online STD risk assessment and testing as well as online counselling and education could enhance opportunities for low-threshold service provision. Online services appealed to youth’s needs for convenience, privacy, as well as expedient access to testing and/or counselling [15]. However, youth also appear to have relatively low tolerance for technologies that they perceive to be antiquated (e.g., printing lab requisition forms, or e-mail, perceived by most participants as not fitting well with the realities of their generation’s information-seeking preferences, primarily because e-mail lacks expediency) [15].

Interestingly, evidence also suggests that certain health behaviours might spread through social ties, of which online social networks are one example. Social network analysis is therefore regarded as a promising, new field for monitoring the spread of STDs, and for development of new interventions for STD control. Actually, online social networking sites, like Facebook, Twitter, MySpace, LinkedIn, etc.… are being used for sexual health promotion [13], but despite their assumed potential benefits to disseminate online public health interventions, there is a lack of knowledge regarding the effectiveness of those approaches [13].

The use of social media in health promotion therefore be valued for their potential to engage with young audiences; in fact, a recent study concluded that more than 50% of the teenagers disclosed information about health risk behaviours in social media public profiles like MySpace [16]. Consequently, social networks appear to be fertile ground for gathering information about young people’s health behaviours and attitudes.

### Use of avatars in online health settings

Avatars, also known as player or game characters, are defined in computing terms as the graphical representation of a user. But in psychological terms represents much more: gives anonymity and freedom to users (facilitating their access to sensitive information) and at same time represents an opportunity for changing the behaviour of users [17] due to its potential to serve as models. Every day, millions of users interact in real-time via avatars in online environments and these avatars or virtual self can even be programmed to behave independently of the physical self, maximizing its utility as a persuasive agent/health behaviour modelling [18,19].

Related to the behaviour of users when defining the characteristics of the avatars, while some authors have been analysing the importance of realism and interactivity of avatars in terms of behaviour and form on e-learning environments [20-24] other researchers have studied the characteristics of avatars in relation to the context [25] and have found that in the dating and gaming treatments participants accentuated certain aspects of their avatar to reflect the tone and perceived expectations of the context. For instance, avatars in dating were made to look more attractive while avatars in gaming were made to look more intellectual [25].

Studies have shown that people infer their expected behaviours and attitudes from observing their avatar’s appearance, this phenomenon is known as the Proteus effect [17]. These studies also analysed the expression of personality through avatars [26] and the relation between virtual and real world and have shown how the appearance of these avatars (i.e., digital representations of ourselves) can lead to behavioural changes in users, changing both how people interact with others in avatar-based online communities as well in subsequent face-to-face interactions [17,27].

The main objective of this project is to achieve that North Norwegian youngsters become more aware of STDs through the use of popular technologies among young people.

## Methods

To achieve the main objective a Virtual Clinic for Sexually Transmitted Diseases (VCSTD) will be developed. The VCSTD, which will be hosted at http://www.sjekkdeg.no, will provide early guidance and reliable information sources concerning reproductive health, delivered in a novel and innovative way to the younger population. The VCSTD consists of an “avatar” supported intervention in a serious gaming like and e-learning environment, which will bypass direct physical access (in person) to reliable medical information, as well as allowing the youngsters to share that information in social media, and thus helping the VCSTD to be disseminated to more people.

The VCSTD will be developed under the premise that Information and Communication Technology may empower younger citizens to change their health related behaviour and lifestyle. ICT supported, health related tasks such as seeking medical information could have a large effect in the reduction of costs and also allow reaching more people to provide behavioural intervention and support at less financial and personnel costs [14]. The VCSTD will also incorporate a feature to allow users to download and post content from the VCSTD in their Facebook profiles or other social media networks; and a geolocation service that can be enabled/disabled voluntarily by users, and that may provide information, for example: where to find condoms or the location of the nearest health center.

The VCSTD it is expected to start at the end of August 2012.

### Users’ involvement

Youngsters from the two northernmost regions in Norway, Troms and Finnmark are expected to participate in this project. But due to the VCSTD project will be disseminated through Facebook and users could share it in the social media, participants from other parts of Norway could be found, or even Norwegian-speakers from/in other countries.

Upon registration, which consists of creating an avatar (username, which is the name of the avatar the user created, password and secret question), the participant will receive a web unique link which enable him/her to access the secured web and to enter data. To access the VCSTD the user has two possibilities: 1. Login by writing the username and password, 2. Clicking on the link and writing the password. If the user needs to recover his/her avatar web link and or password, the combination of giving the username and answering the secret question can be used.

Then, users could enter into the virtual clinic after choosing an avatar to represent them anonymously. Inside the clinic they will have access to a set of reproductive health resources such as videos, articles, audio, quizzes and links. Each participant will be asked to interact more than one time, although every created avatar from an IP belonging to one of the two northernmost regions (Troms or Finnmark) will be analysed.

Both, the avatar and the provided data will be voluntarily entered by each participant and will be stored in their own profile. The stored information will consist in different quizzes about his/her knowledge on STD and how to prevent it (eg. “How much I know about Chlamydia”). Other collected information will be the answers on a symptom check list (according to *Norsk Dermatologisk Selskaps*).

### Techniques for recruitment, gathering and managing data

Users will be recruited in two different “channels”, both targeting North Norwegian youngsters. Recruitment channel 1 will utilize social media for recruitment (e.g., Facebook), while recruitment channel 2 will be targeting traditional venues for public health recruitment (e.g., newspapers, static online information ads). Recruitment to the VCSTD will be aimed specifically at the two northernmost regions in Norway. This will include utilization of social media/networks. The trial will run one year from start-up.

Due to the nature of the project identifying individual participants will not be possible. Data analyses will therefore be conducted on publically available health data relevant to STDs in Troms and Finnmark: 1) Absolute number of chlamydia tests; 2) Amount of emergency contraception medication sold ("Postinor", "Norlevo", "Ellaone"); and 3) Number of abortions.

Also, experiences of usefulness will be explored through participants’ voluntary responses to a feedback form available in the VCSTD. The usage data of the system will be also used to evaluate the usability of the system (average time spent in the system by the users).

Users of the VCSTD will be issued with an invitation to answer voluntarily one or more *adhoc* and anonymous questionnaires to assess how the site has affected their knowledge on sexual health, STD and specifically Chlamydia, and possibly their behaviour change, and/or motivation to test themselves for STDs.

Users could be asked also to answer voluntarily and anonymous short personality inventories, like the Sensation-Seeking Scale [28] or the Locus of Control Scale [29].

A review of the project at the Regional committee for medical and health research ethics (REK Nord) is not applicable as patients are not involved in the research, and all health data investigated are anonymous.

### Analysis

The purpose of the study is descriptive, so the results will be expressed in form of frequencies and percentages for each categorical variables and mean, standard deviation (SD) and 95% confidence interval (95% CI) for continuous variables. T-Tests, ANOVA, correlation and Chi-Square analyses will be performed as well. The opinions of users in the *adhoc* questionnaires about the site and how it has affected their knowledge will be analysed using qualitative techniques.

Quantitative data analysis will be performed with the SPSS statistical package version 19.

## Discussion

This study will examine the usefulness of an online public health intervention avatars based and using computer/smartphone mediated communication and social networking in prevention of sexually transmitted diseases and healthier sexual practices among North-Norwegian youngsters.

Considering that Norway has one of the highest Internet penetration rates in the world (97.2%) [8] and these media are among the most powerful influences in the lives of young people [9], a virtual clinic on STD accessible through different technological platforms (for instance laptop, smartphone, tablet computer) could represent a good way for educating young people on preventing STDs, especially Chlamydia. And the avatars of the VCSTD could give anonymity and freedom to users (facilitating their access to sensitive information) and at same time could represent an opportunity for changing the sexual behaviour of youngsters [17,30]. Taking into account that 96% of Norwegians aged between 16 and 24 years old are using Internet for social networking purposes (facebook, twitter, etc.) [10], the social media could be a valuable resource for sexual health promotion to youngsters, recruiting users and reach more people.

If the VCSTD shown to be effective, the intervention could prove to be an affordable and widely accessible intervention to decrease risky sexual practices among the younger population.

## Abbreviations

AIDS: Acquired immunodeficiency syndrome; ICT: Information and Communication Technology; STD: Sexually transmitted disease; VCSTD: Virtual clinic for sexually transmitted diseases.

## Competing interest

The authors declare that they have no competing interests.

## Authors’ contributions

EG and JAS have contributed to conception and design, drafting and revising the manuscript critically for important intellectual content. RW and MA have contributed to conception, design and revising the manuscript critically for important intellectual content. All authors have read and approved the final manuscript.

## Pre-publication history

The pre-publication history for this paper can be accessed here:

http://www.biomedcentral.com/1472-6947/12/120/prepub

## References

[B1] Lengen C Jäger S Kistemann T The knowledge, education and behaviour of young people with regard to Chlamydia trachomatis in Aarhus, Denmark and Bonn, Germany: do prevention concepts matter? Soc Sci Med 2010 70 11 1789 1798 Available from: http://www.ncbi.nlm.nih.gov/pubmed/20307923 10.1016/j.socscimed.2010.01.048 20307923

[B2] Mylonas I Female genital Chlamydia trachomatis infection: where are we heading? Arch Gynecol Obstet 2012 285 5 1271 1285 Available from: http://www.ncbi.nlm.nih.gov/pubmed/22350326 10.1007/s00404-012-2240-7 22350326

[B3] World Health Organization Sexually Transmitted Diseases Available from: http://www.who.int/vaccine_research/diseases/soa_std/en/index1.html

[B4] Folkehelseinstituttet-Norwegian Institute of Public Health Genitale chlamydiainfeksjoner i Norge 2009 2010 Available from: http://www.fhi.no/eway/default.aspx?pid=233&trg=Area_5626&MainArea_5661=5618:0:15,1327:1:0:0:::0:0&MainLeft_5618=5626:84463::1:5624:1:::0:0&Area_5626=5544:84495::1:5628:1:::0:0

[B5] Statistics Norway Statistics Norway 2012 Available from: http://www.ssb.no/english/

[B6] Kløvstad H Jakopanec I Blystad H Genitale chlamydiainfeksjoner i Norge 2009 MSIS-rapport 10/2010 (In Norwegian)

[B7] Folkehelseinstituttet Seksuelt overførbare sykdommer 2010 Available from: http://www.fhi.no/eway/default.aspx?pid=233&trg=MainArea_5661&MainArea_5661=6068:0:17,4210:1:0:0:::0:0

[B8] Internet World Stats 2012 European Internet and Population Statistics Available from: http://www.internetworldstats.com/stats4.htm

[B9] Rideout VJ Foehr UG Roberts DF Generation M2. Media in the Lives of 8- to 18-Year-Olds. A Kaiser Family Foundation Study 2010

[B10] Statistics Norway Use of ICT in households 2011 Available from: http://www.ssb.no/ikthus_en/

[B11] Fairley CK Using information technology to control STIs Sex Transm Infect 2011 87 Suppl 2 ii25 ii27 Available from: http://sti.bmj.com/cgi/doi/10.1136/sti.2010.048330 10.1136/sti.2010.048330 22110149 PMC3610391

[B12] Bailey JV Murray E Rait G Mercer CH Morris RW Peacock R Interactive computer-based interventions for sexual health promotion (Review) Cochrane Database Syst Rev 2010 8 9 CD006483 20824850 10.1002/14651858.CD006483.pub2 PMC13139710

[B13] Gold J Pedrana AE Sacks-Davis R Hellard ME Chang S Howard S A Systematic Examination of the Use of Online Social Networking Sites for Sexual Health Promotion BMC Publ Health 2011 11 1 583 Available from: http://www.ncbi.nlm.nih.gov/pubmed/21777470 10.1186/1471-2458-11-583 PMC3155501 21777470

[B14] Swendeman D Rotheram-borus MJ Internet and mobile phone delivery vehicles for global diffusion Curr Opin Psychiatry 2011 23 2 139 44 20087189 10.1097/YCO.0b013e328336656a PMC2881840

[B15] Shoveller J Knight R Davis W Gilbert M Ogilvie G Online Sexual Health Services: Examining Youth’s Perspectives. Can J Public Health 2012 103 1 14 8 22338322 10.1007/BF03404062 PMC6973649

[B16] Moreno MA Parks MR Zimmerman FJ Brito TE Christakis DA Display of Health Risk Behaviors on MySpace by Adolescents Arch Pediatr Adolesc Med 2009 163 1 27 34 10.1001/archpediatrics.2008.528 19124700

[B17] Yee N Bailenson J The Proteus Effect: The Effect of Transformed Self-Representation on Behavior Hum Commun Res 2007 33 3 271 290 Available from: http://doi.wiley.com/10.1111/j.1468-2958.2007.00299.x 10.1111/j.1468-2958.2007.00299.x

[B18] Fox J Bailenson J Virtual Self-Modeling: The Effects of Vicarious Reinforcement and Identification on Exercise Behaviors Media Psychology 2009 12 1 1 25 Available from: http://www.informaworld.com/openurl?genre=article&doi=10.1080/15213260802669474&magic=crossref||D404A21C5BB053405B1A640AFFD44AE3 10.1080/15213260802669474

[B19] Fox J Bailenson JN The use of doppelgängers to promote health behavior change CyberTherapy & Rehabil 2010 3 2 16 17

[B20] van Vugt HC Bailenson JN Hoorn JF Konijn EA Effects of facial similarity on user responses to embodied agents. ACM Transactions on Computer-Human Interaction 2010 17 2 1 27 Available from: http://portal.acm.org/citation.cfm?doid=1746259.1746261

[B21] Bailenson JN Schroeder R The Effect of Behavioral Realism and Form Realism of Real-Time Avatar Faces on Verbal Disclosure, Nonverbal Disclosure, Emotion Recognition, and Copresence in Dyadic Interaction Presence 2006 15 4 359 72 10.1162/pres.15.4.359

[B22] Bailenson JN Yee N Blascovich J Beall AC Lundblad N Jin M The Use of Immersive Virtual Reality in the Learning Sciences: Digital Transformations of Teachers, Students, and Social Context J Learn Sci 2008 17 1 102 141 Available from: http://www.tandfonline.com/doi/abs/10.1080/10508400701793141 10.1080/10508400701793141

[B23] Bailenson J Patel K Nielsen A Bajscy R Jung S-H Kurillo G The Effect of Interactivity on Learning Physical Actions in Virtual Reality Media Psychology 2008 11 3 354 376 Available from: http://www.tandfonline.com/doi/abs/10.1080/15213260802285214 10.1080/15213260802285214

[B24] Given LM Ruecker S Simpson H Sadler E(B) Ruskin A Customizable Avatars for a Health Information System: An Exploratory Design J American Soc Information Sci Technology 2007 58 11 1610 1617 Available from: http://doi.wiley.com/10.1002/asi.20645 10.1002/asi.20645

[B25] Vasalou A Joinson AN Me, myself and I: The role of interactional context on self-presentation through avatars. Computers in Human Behavior 2009 25 2 510 520 Available from: http://linkinghub.elsevier.com/retrieve/pii/S0747563208002069 10.1016/j.chb.2008.11.007

[B26] Yee N Harris H Jabon M Bailenson JN The Expression of Personality in Virtual Worlds Soc Psychol Pers Sci 2011 2 5 12 10.1177/1948550610379056

[B27] Yee N Bailenson JN The difference between being and seeing: the relative contribution of self-perception and priming to behavioral changes via digital self-representation Media Psychol 2009 12 2 195 209 Available from: http://www.tandfonline.com/doi/abs/10.1080/15213260902849943 10.1080/15213260902849943

[B28] Zuckerman M Kolin EA Price L Zoob I Development of a sensation-seeking scale J Consult Psychol 1964 28 6 477 82 14242306 10.1037/h0040995

[B29] Rotter JB Internal versus external control of reinforcement: a case history of a variable Am Psychol 1990 45 4 489 93

[B30] Yee N Bailenson JN Ducheneaut N The Proteus Effect: Implications of Transformed Digital Self-Representation on Online and Offline Behavior Communication Res 2009 36 2 285 312 10.1177/0093650208330254

